# Selection and Validation of Stable Reference Genes for RT-qPCR Analyses of *Rumex patientia* (Polygonaceae) Under Four Abiotic Stresses

**DOI:** 10.3390/genes16070787

**Published:** 2025-06-30

**Authors:** Qian Yang, Xiaoli Li, Rongju Qu, Yuping Liu, Xu Su, Jiarui Jin, Mingjun Yu, Zhaxi Cairang, Penghui Zhang, Yinghui Zheng, Xuanlin Gao, Marcos A. Caraballo-Ortiz

**Affiliations:** 1 School of Life Sciences, Qinghai Normal University, Xining 810008, China; yq20011223@163.com (Q.Y.); lxl13369714361@163.com (X.L.); qu203462@163.com (R.Q.); jin13546511269@163.com (J.J.); 15234934641@139.com (M.Y.); cairangzhaxi161915@163.com (Z.C.); zph200720@163.com (P.Z.); yhzheng99@163.com (Y.Z.); gxl202407@163.com (X.G.); 2 Key Laboratory of Biodiversity Formation Mechanism and Comprehensive Utilization of the Qinghai-Tibet Plateau in Qinghai Province, Qinghai Normal University, Xining 810008, China; 3 Academy of Plateau Science and Sustainability, Qinghai Normal University, Xining 810016, China; 4 Department of Botany, National Museum of Natural History, Smithsonian Institution, Washington, DC 20013, USA; caraballom@si.edu; 5 Department of Biology, University of Mississippi, Oxford, MS 38677, USA

**Keywords:** *Rumex patientia*, candidate reference genes, RT-qPCR, abiotic stress, validation

## Abstract

**Background**: *Rumex patientia* (Polygonaceae), a perennial herbaceous species predominantly found in northern temperate regions, has been historically utilized in traditional Chinese medicine for its hematological regulatory properties, including blood cooling, hemostasis, and detoxification. Despite the pharmacological value of this species, unvalidated reference genes compromise precise gene expression profiling. **Methods**: We initially selected eight candidate genes (*ACT*, *GAPDH*, *YLS*, *SKD1*, *UBQ*, *UBC*, *EF-1α*, *TUA*) from *R. patientia* transcriptomes and then assessed their transcriptional stability using RT-qPCR across root, stem, and leaf tissues under four abiotic stresses: cold, drought, salinity, and heavy metal exposure. **Results**: *ACT* emerged as the most stable reference gene in three specific scenarios: root/leaf tissues under cold stress, stems during drought exposure, and roots subjected to salt treatment, revealing distinct tissue–stress response patterns. *TUA* emerged as the most stable reference in cold- and salt-challenged stems, while *SKD1* exhibited superior stability in drought-affected roots/leaves, heavy-metal-stressed tissues, and salt-treated leaves. Validation via the drought-inducible MYB transcription factor confirmed reference gene reliability. **Conclusions**: This work pioneers a standardized reference gene framework for *R. patientia* under multi-stress conditions, offering essential methodological foundations for subsequent molecular research in this medicinal plant.

## 1. Introduction

Plants growing in unstable environments frequently encounter biotic stresses throughout their life cycle [1]. Abiotic stressors, including cold, frost, heat, drought, soil salinity, and heavy metal toxicity, significantly constrain plant growth and agricultural productivity globally [2]. Notably, cold, drought, and soil salinity critically define plant biogeography, constrain crop yields, and jeopardize global food security [3]. Heavy metal stressors, however, trigger intricate biochemical disturbances that compromise plant survival. These toxic elements impair enzymatic functions, displace vital metal cofactors in biomolecules, and generate reactive oxygen species [4]. Such molecular disturbances ultimately destabilize cellular membranes and disrupt fundamental metabolic processes, including photosynthetic pathways, energy production, and ion regulation. To counter these pressures, plants evolve adaptive mechanisms to minimize cellular damage [5].

A comprehensive evaluation of plant responses to abiotic stressors necessitates an analysis of gene expression dynamics [6]. While conventional approaches like Northern blot and competitive quantitative PCR have historically dominated transcript profiling, these techniques face limitations including narrow dynamic ranges, insufficient sensitivity, prolonged processing times, and substantial RNA requirements [7,8]. In contrast, real-time quantitative PCR (RT-qPCR) has become a standard methodology due to its enhanced sensitivity, precision, rapid throughput, and capacity for absolute mRNA quantification [9]. Yet its accuracy remains vulnerable to multiple technical variables, including RNA integrity, enzymatic fidelity during reverse transcription, and amplification consistency [10]. Consequently, implementing normalization using endogenous reference genes is essential for reliable data [11]. Reference genes should exhibit invariant expression across developmental stages and tissue types while resisting experimental perturbations [12]. Commonly employed plant reference genes include *Actin*, *GAPDH*, *EF-1α*, *β-tubulin*, and *18S rRNA* [13]. These genes typically display reduced variability relative to alternative references. Despite their prevalent usage, prior research necessitates taxon- and condition-specific reference gene selection for experimental rigor [7].

*R. patientia,* an Asian–European perennial herb (Polygonaceae), features erect stems and robust roots while being pharmacologically significant [14,15]. Some of these properties include detoxification, laxative, blood-activating, heat-clearing, hemostasis, and insecticidal properties. Current studies on *R*. *patientia* have explored multiple aspects of its biology and physiology, including the characterization of its chemical constituents [16,17,18], the composition of rhizosphere and non-rhizosphere bacterial communities [19], the anti-inflammatory effects of root extracts [20], the physiology of stomata [21], the effect of ultraviolet light on leaf development [22], a transcriptomic analysis of cold stress [23], and its genetic diversity across habitats [24]. Demirezer et al. isolated emodin-6-O-β-D-glucopyranoside and flavan-3-ol (6-chlorocatechin) from *R. patientia*, confirming their cytotoxic activity and radical scavenging capacity in antioxidant studies [15]. An assessment of transcriptome data detected 66 up-regulated genes related to cold stress in *R. patientia*, representing the *MYB*, *AP2*/*ERF*, *CBF*, *Znf*, *bZIP*, *NAC*, and *COR* families [23]. Despite extensive research, validated reference genes remain undocumented for *R. patientia*, crucially constraining mechanistic insights into its gene regulatory networks.

Eight candidate reference genes were identified from *R. patientia* transcriptomes: *ACT*, *GAPDH*, *YLS*, *SKD1*, *UBQ*, *UBC*, *EF-1α*, and *TUA*. Their expression stability was evaluated under four abiotic stresses via multi-algorithm validation (ΔCt, BestKeeper, geNorm, NormFinder, RefFinder). Optimal and suboptimal references were further validated using the drought-responsive *MYB* gene. This work establishes a standardized reference gene framework for *R. patientia* across tissues and stress conditions, enabling reliable RT-qPCR normalization in related *Rumex* species.

## 2. Materials and Methods

### 2.1. Plant Materials and Experimental Treatments

Mature *R. patientia* seeds were harvested from wild populations in Nangqian County, Yushu Tibetan Autonomous Prefecture, Qinghai Province (32°16′55.8″ N, 96°27′39.6″ E). Plump, uniformly sized, and disease-free seeds were selected. Followed rinsing with distilled water, germination was initiated on moist filter paper in Petri dishes. Resultant seedlings underwent cultivation in growth chambers (26 °C; 16/8 h light/dark photoperiod). After one month, seedlings exhibiting uniform size and development (four-leaf stage) were selected for subsequent experiments.

Four abiotic environmental stresses that could affect *R. patientia*, namely cold, soil salinity, heavy metal contamination, and drought, were selected. For the cold treatment, we exposed *R. patientia* seedlings to constant cold stress by placing them in a refrigerator set at 4 °C. The salt and drought treatments were simulated via watering each pot with 300 mL of 200 mM NaCl solution and 300 mL of 10% (*w*/*v*) PEG-6000 (polyethylene glycol, molecular weight 6000), respectively. For the heavy metal treatment, we watered the pot soils using 300 mL of 200 mM CdCl_2_ solution. At 0, 6, 12, 24, and 48 h post-treatment, triplicate samples of roots, stems, and leaves were collected, immediately flash-frozen in liquid nitrogen, and stored at −80 °C for subsequent molecular analysis.

### 2.2. RNA Extraction, Quality Assessment, and cDNA Synthesis

Total RNA was isolated from root, stem, and leaf tissues with a Plant Total RNA Extraction Kit (TaKaRa, Dalian, China). Purity/concentration was measured spectrophotometrically (NanoDrop 2000, Thermo Fisher Scientific, Waltham, MA, USA) at A260/A280 (1.90–2.10). Integrity was verified via 1.0% agarose gel electrophoresis. cDNA synthesis used a gDNA-eliminating reverse transcription kit (PrimeScript RT, TaKaRa). cDNA products were 10× diluted in nuclease-free water and stored at −20 °C for RT-qPCR analyses.

### 2.3. Reference Gene Selection and Primer Design

The *R. patientia* transcriptome (NCBI SRA: SRR18011456) was used to screen eight candidate reference genes: *ACT*, *GAPDH*, *YLS*, *SKD1*, *UBQ*, *UBC*, *EF-1α*, and *TUA*. Complete ORFs were annotated, and Primer Premier 5.0 (Ding et al., 2004) was used to design gene-specific primers with the following criteria: melting temperature (Tm) 54–62 °C, primer length 18–25 bp, GC content 40–60%, and amplicon size under 300 bp. The specificity of the primers was confirmed via 1% agarose gel electrophoresis, Sanger sequencing of purified PCR products (performed by BGI Genomics with bidirectional primers), and melting curve analysis. All amplified sequences were aligned to the *R. patientia* transcriptome reference sequences using SnapGene 6.0.2 software to confirm primer specificity and target identity. We determined amplification efficiency (E) and correlation coefficients (R^2^) via standard curves created with ten-fold serial dilutions (Table 1).

### 2.4. Real-Time Quantitative PCR Analysis

Using a QuantStudio^TM^ 6 Flex System (Thermo Fisher Scientific, Waltham, MA, USA) and TB Green chemistry, RT-qPCR reactions (20 µL total volume) contained the following components: 10 µL TB Green Premix Ex Taq, 0.4 µL each PCR primer, 0.4 μL ROX Reference Dye (50×), 6.8 μL ddH_2_O, and 2 μL cDNA. Thermal cycling conditions were as follows: 95 °C for 30 s and then 48 cycles of 95 °C for 5 s and 58 °C for 34 s.

### 2.5. Gene Expression Stability Analysis

The stability of gene expression was evaluated using a multi-algorithm strategy that combined four computational tools: the ΔCt method, geNorm v3.5 [25], NormFinder v20 [26], and BestKeeper v1 [27]. This integrated analysis was performed in conjunction with the RefFinder web platform [28]. RT-qPCR generated raw quantification cycle (Cq)/cycle threshold (Ct) values for the assessment. For the ΔCt method, pairwise Ct differences between all candidate genes were computed across all samples, followed by the calculation of the average standard deviation for each gene, where lower values indicate higher stability [29]. The Ct values were converted to 2−ΔCt using the following formula: ΔCt = sample Ct − minimum Ct. These transformed values were input into geNorm to compute expression stability metrics (M-values), with lower M-values indicating higher transcriptional stability [30]. Pairwise variation (V*_n_*/V*_n+_*_1_) identified the appropriate number of reference genes (n) as the smallest number where V*_n_*/V*_n+_*_1_ ≤ 0.15. NormFinder identifies the optimal normalization gene within a candidate set by evaluating and ranking genes based on expression stability across samples and experimental conditions; the gene with the lowest stability value exhibits the most stable expression. In this context, the gene with the lowest stability value is deemed to have the most stable expression among the examined gene set. Raw Ct values were used to calculate the coefficient of variance (CV) and standard deviation (SD) of candidate genes in BestKeeper. Integrative analysis was performed using RefFinder to synthesize multi-algorithm datasets, incorporating geNorm-derived M-values, NormFinder stability indices, BestKeeper variability metrics (CV/SD), and ΔCt normalization factors. Through weighted geometric mean computations [31], this approach generated consensus stability rankings, ensuring statistically robust reference gene selection across methodological frameworks.

### 2.6. Reference Gene Validation

RefFinder’s comprehensive ranking framework was utilized to validate reference gene selection efficacy, identifying both optimal and suboptimal candidates under drought stress. The drought-responsive MYB transcription factor family—renowned for its phylogenetic prominence in plant stress adaptation—served as a validation target. *MYB* genes orchestrate transcriptional regulation of developmental processes, phytohormone signaling networks, and stress response pathways, exhibiting characteristic drought-inducible expression patterns [32]. Using RT-qPCR with the forward primer 5′-ATCCTAAGCAAGAGCCAAGGTCAC-3′ and the reverse primer 5′-TACCTCTCAACCCCAAGAAATCAT-3′, *MYB* gene amplification enabled the analysis of its expression across *R. patientia* tissues under drought stress. Expression quantification used the 2^−ΔΔCT^ method, employing the worst and top two RefFinder-pinpointed reference genes per stressed tissue.

## 3. Results

### 3.1. RNA Integrity and Primer Specificity

**Figure 1:**
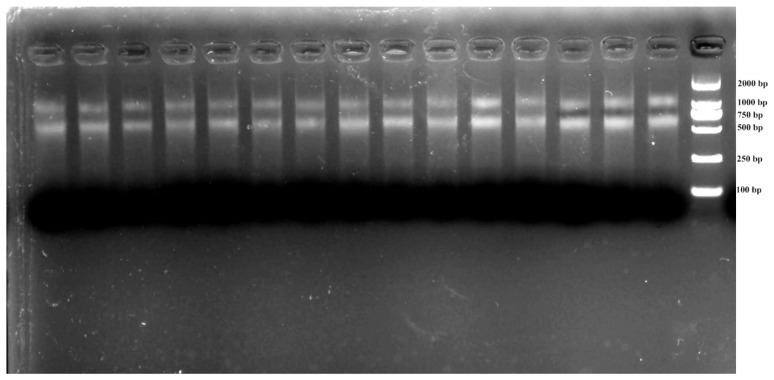
Assessment of RNA integrity by partial agarose gel electrophoresis.

**Figure 2:**
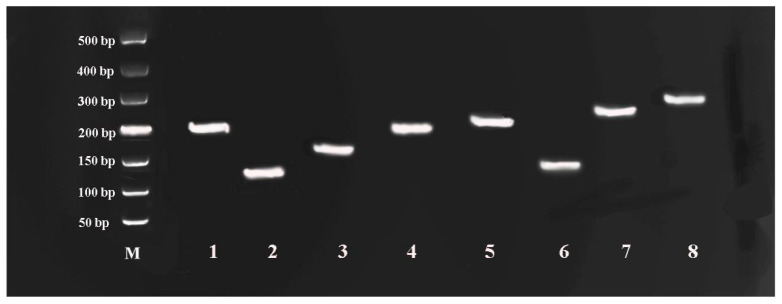
Amplicon size of eight candidate reference genes of *R. patientia*. Numbers 1 to 8 correspond to genes *ACT*, *GAPDH*, *YLS*, *SKD1*, *UBQ*, *UBC*, *EF-1α*, and *TUA*, respectively. M represents a 500 bp DNA ladder.

**Figure 3:**
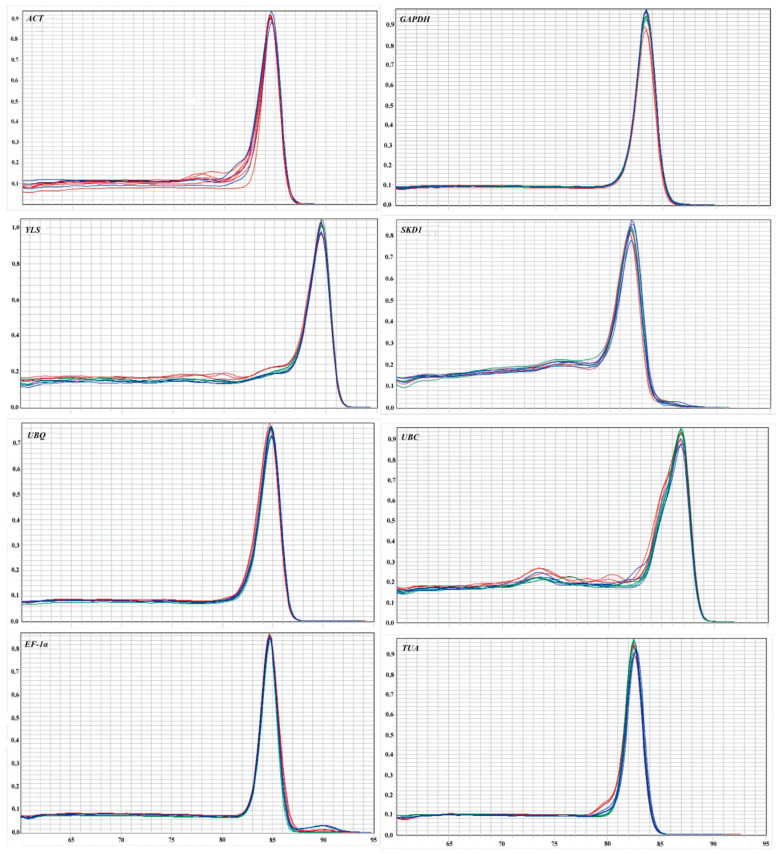
Melting curves showing primer specificity of eight candidate reference genes of *R. patientia*.

**Figure 4:**
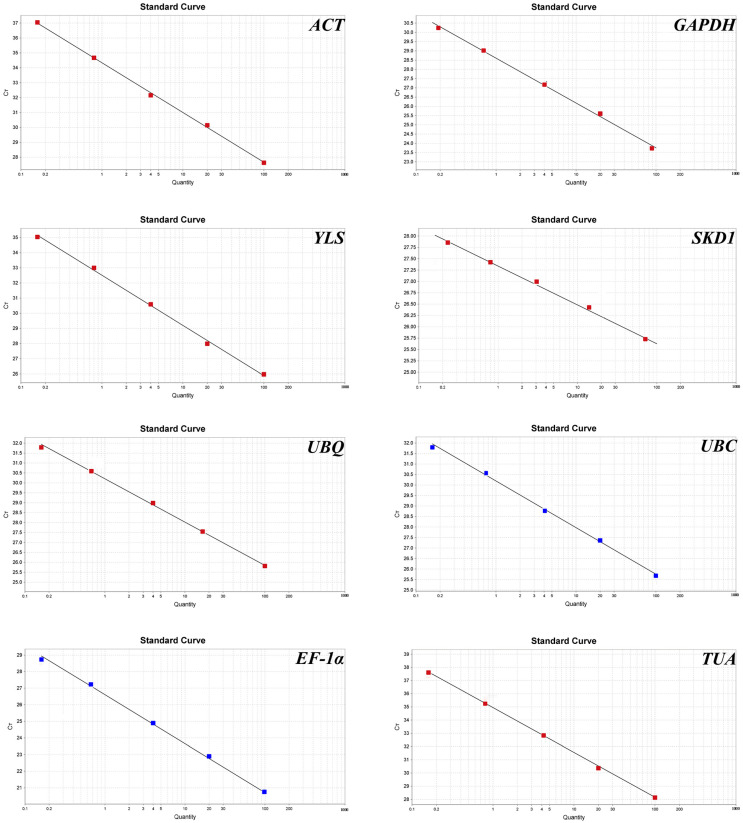
Standard curves of eight candidate reference genes of *R. patientia* based on 10-fold dilution of cDNA.

Total RNA isolation across samples yielded A_260_/A_280_ absorbance ratios (1.9–2.1) and sharp 18S/28S bands (Figure 1), confirming suitability for RT-qPCR analysis. Primer specificity was confirmed through three-tier validation: (1) target-specific electrophoretic bands (Figure 2), (2) monophasic melt curve profiles (Figure 3), and (3) amplification efficiency parameters. Quantitative analysis revealed primer performance metrics within acceptable thresholds—amplification efficiencies spanned 92.6–127.4% (*UBC* to *GAPDH*), and regression coefficients (R^2^) varied from 0.97 (in *GAPDH*) to 0.99 (in *ACT*) (Figure 4, Table 1).

### 3.2. Expression Profile of Reference Genes

**Figure 5:**
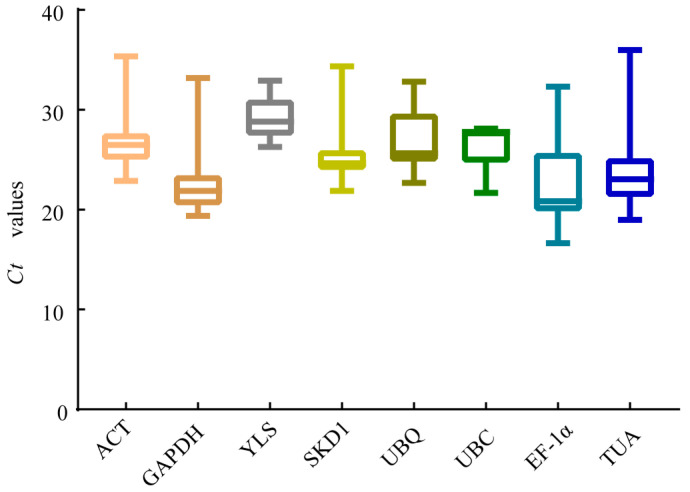
Mean Ct values of candidate reference genes in *R. patientia* across all treatments. The box plot displays the Ct value distribution: top line (upper quartile), middle line (median), bottom line (lower quartile), top edge (maximum), and bottom edge (minimum).

RT-qPCR analysis typically requires Ct values within 15–35, as proposed by prior research [33]. In this study, all samples tested showed Ct values from 16.66 to 35.97. Among them, *EF-1α*, *GAPDH*, and *TUA* showed the highest expression (mean Ct: 22.84, 23.12, 24.18), while *YLS* and *ACT* had the lowest (29.31 and 27.07, respectively) across treatments. Most candidate genes showed broad Ct variation across tissues/treatments, while *UBC* and *YLS* exhibited relatively stable expression in *R. patientia* under all conditions (Figure 5).

### 3.3. Expression Stability of Candidate Reference Genes

Reference gene stability was assessed using five approaches: geNorm, NormFinder, BestKeeper, RefFinder, and the Delta-Ct method. Differential outcomes emerged across Ct data treatments, as subsequently detailed.

#### 3.3.1. The Comparative Delta-Ct Method Analysis

**Figure 6:**
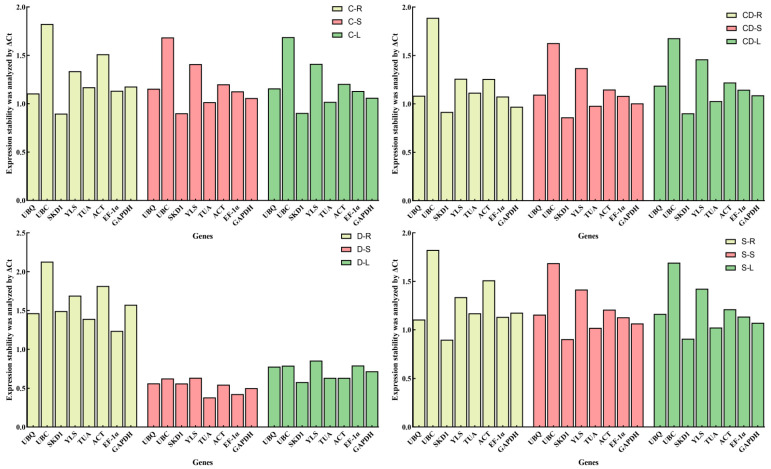
Stability assessment of *R. patientia* reference genes across four stress conditions via ΔCt method. Note: C: low-temperature stress; D: drought stress; CD: heavy metal stress; S: salt stress. R, S, and L stand for root, stem, and leaf, respectively.

The Delta-Ct method evaluates reference gene stability by calculating pairwise differences in Ct values (ΔCt) between all candidate genes across all samples. For each gene pair, the mean ΔCt value and standard deviation (SD) are computed. These pairwise standard deviations are then aggregated for every individual gene, and the mean standard deviation is calculated per candidate gene. Genes demonstrating lower mean standard deviation values exhibit higher expression stability. Under cold stress, *SKD1* exhibited the lowest mean standard deviation (SD) values in roots, stems, and leaves (0.8984, 0.9013, and 0.9044, respectively), while *UBC* showed the highest values (1.8240, 1.6853, and 1.6883), indicating *SKD1* was the most stable reference gene and *UBC* the least stable. Similarly, under heavy metal stress, *SKD1* displayed the lowest SD values across tissues (roots: 0.9169; stems: 0.8610; leaves: 0.9031), whereas *UBC* had the highest (roots: 1.8890; stems: 1.6280; leaves: 1.6785), confirming *SKD1* as the most stable and *UBC* as the least stable reference gene. During drought stress, *EF-1α*, *TUA*, and *SKD1* were the most stable reference genes in roots, stems, and leaves, respectively, with *UBC* being the least stable in roots and *YLS* least stable in stems and leaves. Likewise, under salt stress, *SKD1* demonstrated the highest stability across all tissues, while *UBC* remained the least stable reference gene (Figure 6).

#### 3.3.2. geNorm Analysis

**Figure 7:**
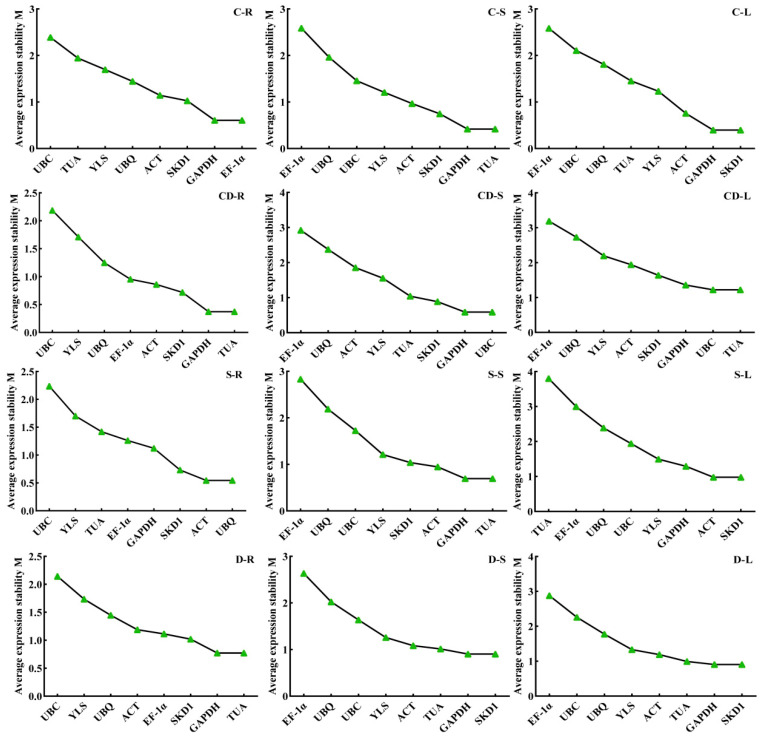
geNorm evaluation of reference gene expression stability in *R. patientia*. Abbreviations in the panels are as follows: C: low-temperature stress; D: drought stress; CD: heavy metal stress; S: salt stress.

**Figure 8:**
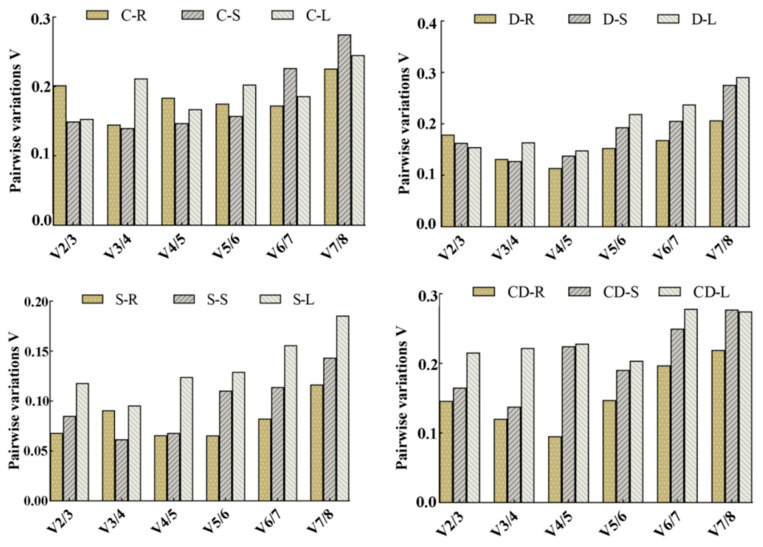
Number of best candidate genes of *R. patientia* analyzed by geNorm. Note: C: low-temperature stress; D: drought stress; CD: heavy metal stress; S: salt stress. R, S, and L stand for root, stem, and leaf, respectively. V*_n_*/V*_n+_*_1_ determines the optimal count of reference genes for data normalization under given experimental conditions.

geNorm analysis revealed tissue-specific stable reference gene pairs under different stresses: for cold stress, roots used *GAPDH* and *EF-1α*, stems used *GAPDH* and *TUA*, and leaves used *GAPDH* and *SKD1*; under drought stress, roots used *GAPDH* and *TUA* while both stems and leaves used *GAPDH* and *SKD1*; for heavy metal stress, the optimal pairs differed across tissues, including *GAPDH* and *TUA*, *GAPDH* and *UBC*, or *UBC* and *TUA*; for salt stress, roots used *ACT* and *UBQ*, stems used *GAPDH* and *TUA*, and leaves used *ACT* and *SKD1*, as shown in Figure 7. Pairwise variation V*_n/n+1_* analysis demonstrated that two reference genes adequately normalized salt-stressed tissues, with V_2/3_ values remaining below the 0.15 threshold. However, for stems subjected to cold, drought, or heavy metal stress, V_2/3_ values exceeded 0.15 while V_3/4_ values remained below 0.15, necessitating the top three reference genes for precise normalization (Figure 8).

#### 3.3.3. NormFinder Analysis

**Figure 9:**
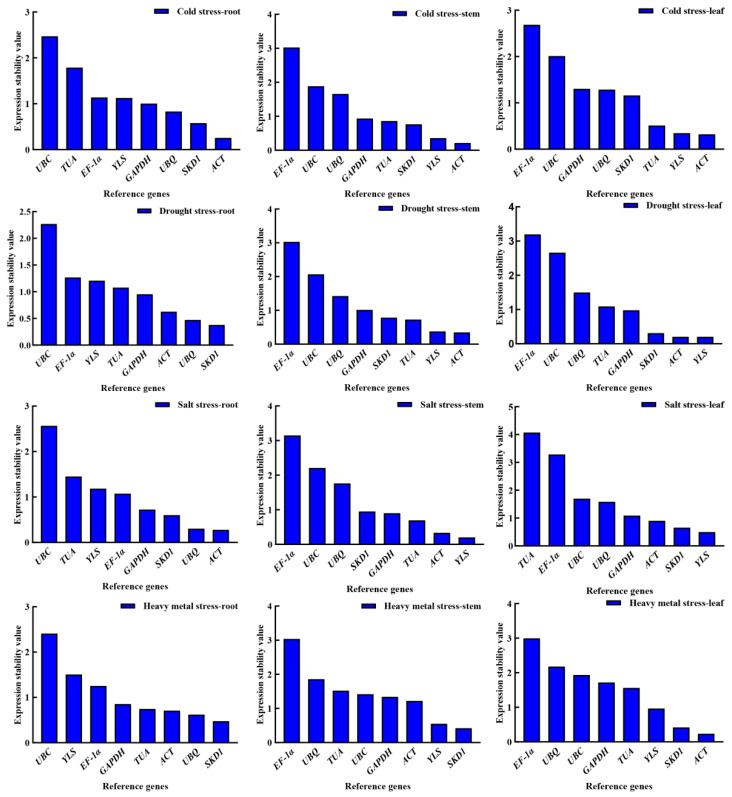
NormFinder stability assessment of candidate reference genes in *R. patientia*.

NormFinder analysis revealed the optimal reference genes across stress conditions: under cold treatment, *ACT* and *SKD1* in roots, *ACT* and *YLS* in stems and leaves; under drought, *SKD1* and *UBQ* in roots, *ACT* and YLS in stems and leaves; for salt stress, *ACT* and *UBQ* in roots, *ACT* and *YLS* in stems, and *YLS* and *SKD1* in leaves; and under heavy metal stress, *SKD1* and *UBQ* in roots, *SKD1* and *YLS* in stems, and *ACT* and *SKD1* in leaves (Figure 9).

#### 3.3.4. BestKeeper Analysis

Using BestKeeper, we assessed the stability of reference genes through CV and SD. Reference genes with the lowest CV and SD were considered the most stable. BestKeeper results showed that under salt treatment, the most stable genes in roots, stems, and leaves were *UBC* (1.75 ± 5.81), *SKD1* (0.36 ± 1.48), and *SKD1* (0.71 ± 2.89), respectively. In drought treatment, the most stable genes were *UBC* (0.2 ± 0.73) in roots, *SKD1* (0.36 ± 1.48) in stems, and *GAPDH* (0.45 ± 2.21) in leaves. For cold treatment, the most stable genes were *UBC* (0.25 ± 0.92) in roots, *SKD1* (0.46 ± 1.81) in stems, and *GAPDH* (0.97 ± 4.67) in leaves. In heavy metal treatment, the most stable genes were *UBC* (0.19 ± 0.68) in roots, *SKD1* (0.46 ± 1.81) in stems, and *SKD1* (0.4 ± 1.63) in leaves (Table 2).

#### 3.3.5. RefFinder Analysis

**Figure 10:**
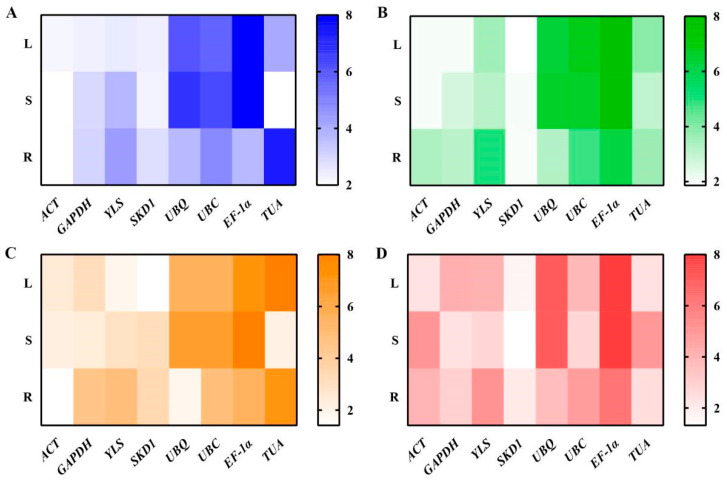
Heatmap visualization of RefFinder-based expression stability for eight reference genes in *R. patientia*. Note: (**A**) Cold stress, (**B**) drought stress, (**C**) salt stress, (**D**) heavy metal stress. R, S, and L denote root, stem, and leaf, respectively.

Comprehensive gene stability rankings revealed that under cold treatment, the most stable genes were *ACT* and *SKD1* in roots, *ACT* and *TUA* in stems, and *ACT* and *GAPDH* in leaves, with *TUA* and *EF-1α* being the least stable. During drought treatment, roots showed *SKD1* and *GAPDH* as the most stable, while stems and leaves both had *ACT* and *SKD1* as optimal, and *EF-1α* was the least stable. For heavy metal treatment, *ACT* and *SKD1* were the most stable in roots, *SKD1* and *GAPDH* in stems, and *SKD1* and *TUA* in leaves, again with *EF-1α* as the least stable. Under salt stress, *ACT* and *UBQ* were the most stable in roots, *TUA* and *ACT* in stems, and *SKD1* and *YLS* in leaves, while *EF-1α* and *TUA* were the least stable, as shown in Figure 10.

### 3.4. Validation of the Stability for Candidate Reference Genes Based on MYB

**Figure 11:**
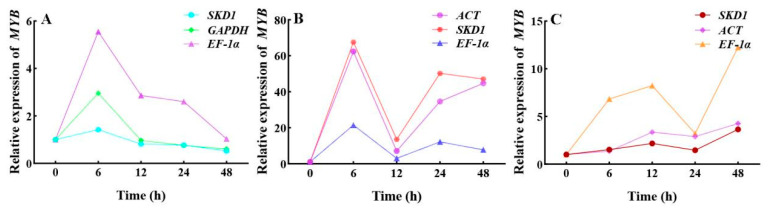
The relative expression of the *MYB* gene in *R. patientia* under drought stress across different tissues. (**A**), (**B**), and (**C**) represent roots, stems, and leaves, respectively.

To validate reference gene stability, *MYB* expression under drought stress was analyzed. Using the stable genes *SKD1* and *GAPDH* in roots yielded significantly different *MYB* expression levels compared to unstable *EF-1α*. Similarly, in stems, the stable pair *ACT* and *SKD1* produced higher *MYB* expression than *EF-1α*. In leaves, unstable *EF-1α* generated values significantly diverging from stable genes *SKD1* and *ACT*, as illustrated in Figure 11.

## 4. Discussion

This study systematically evaluated the stability of eight candidate reference genes (*ACT*, *TUA*, *EF-1α*, *GAPDH*, *UBQ*, *UBC*, *SKD1*, *YLS*) across *R. patientia* tissues (roots, stems, and leaves) under four abiotic stressors. Our results showed that gene amplification efficiency (E) for all genes was within the normal range, fluctuating from 90% to 110% [34], except for *GAPDH*, whose E value reached up to 127%. Despite such anomalies typically compromising expression quantification accuracy [35]), *GAPDH* demonstrated exceptional stability as a reference gene in cold-stressed leaves and drought-exposed roots. In fact, *GAPDH* performed similarly to *SKD1*, which was the first-ranked internal reference gene, in inferring expression levels of *MYB* in stems under drought stress. Therefore, we inferred that the presence of inhibitors or pipetting errors can explain the amplification efficiency being greater than 100% [36], but these errors had a limited effect on *GAPDH*. However, this gene and protocols for its amplification in *R. patientia* merit further exploration in future studies.

Quantitative cycle threshold values (Ct values) in RT-qPCR reflect gene expression levels, with lower values representing higher gene expression levels [37]. Our comparison of Ct values from eight candidate reference genes in *R. patientia* showed that they ranged from 16 to 36. *TUA* showed the greatest variability in expression abundance. Given these differences, we further analyzed the Ct values obtained in the experiment using the ΔCt method, geNorm, NormFinder, and BestKeeper. Differences in calculation principles among these three software packages explain different results from the same sample (Table 3). Such is the case with gene *SKD1*, which was the most stable in stems under drought stress based on scores from geNorm and BestKeeper, but the fourth most stable in NormFinder. Using the ΔCt method, it was found that *SKD1* demonstrated the most stable expression under cold, salt, and heavy metal stress conditions. This discrepancy likely arises from the distinct algorithms employed: geNorm and BestKeeper primarily assess the pairwise variation or correlation between genes, potentially favoring co-regulated genes like *SKD1* under specific conditions. In contrast, NormFinder explicitly incorporates estimates of both intra- and inter-group variation, making it more sensitive to systematic expression differences across experimental groups, which may have affected *SKD1*’s ranking. Similarly, the most stable reference gene in roots under salt stress was *UBC* based on BestKeeper, but this gene was ranked eighth in geNorm and NormFinder. BestKeeper heavily relies on the correlation of each gene’s expression with the geometric mean of all candidates. A gene like *UBC,* showing strong correlation despite potentially high individual variance, might be highly ranked by BestKeeper. However, geNorm, which focuses on minimizing pairwise variation between genes, and NormFinder, which penalizes genes with high variation across groups, may assign *UBC* a low stability rank if its expression exhibits high variance or lacks consistent co-regulation with other candidates within the salt-stressed root samples.

RefFinder functions as an integrative platform rather than a standalone algorithm, synthesizing outputs from geNorm, NormFinder, BestKeeper, and other computational approaches. It employs scoring or ranking mechanisms to holistically evaluate the stability of candidate reference genes [38]. Therefore, our final assessment performed to account for these differences using RefFinder indicated that *ACT* was the gene with the best stability in roots and leaves of *R. patientia* under cold stress, which was consistent with previous results in *Hibiscus esculentus* (Malvaceae) [39]. Regarding the heavy metal stress treatment, *SKD1* was depicted as the best gene in the three tissues of *R*. *patientia*, and it showed a remarkably good stability in roots and leaves under drought stress and leaves under salt stress. Likewise, *SKD1* was also the preferred endogenous reference gene in *Polygonum cuspidatum* (Polygonaceae) under hormonal stress [40] and in *Pyrus serotina* (Rosaceae) [41]. The putative function of *SKD1* is to encode a protein contributing to vacuolar trafficking and maintaining the large central vacuole, which can confer tolerance to stressors like salt and drought [42,43]. Given the context-dependent variability of reference gene stability across tissues, species, and experimental conditions, systematic screening of optimal normalization genes remains essential prior to gene expression quantification. Besides confirming gene stability with RefFinder, we also recommend validating the gene expression stability of the best reference genes like we did here using *MYB* under drought stress [44].

In conclusion, our study highlights the critical importance of selecting appropriate reference genes for accurate RT-qPCR normalization. Our data emphasize the need to perform gene expression analyses with an appropriate reference gene—typically an experimentally tested or housekeeping gene based on its functions—to achieve accurate results [29]. While traditional housekeeping genes (e.g., *ACT*, *EF-1α*, *GAPDH*, *UBQ*) have been widely used, recent studies confirm that their instability varies dramatically across species and experimental conditions [45]. For instance, *ACT* and *UBQ* were stably expressed in *Triticum aestivum* (Poaceae) but not in *Solanum lycopersicum* (Solanaceae) [46,47]. Importantly, recent validations continue to underscore this context-dependency: novel reference genes *COG7* and *TULP6* were identified as superior to traditional choices like HSP90 and S*DH5* across diverse tissues and stresses in *Norway spruce* [48], while in *Phytophthora capsici* interacting with its host, *EF-1α* and *WS21* were validated as highly stable, whereas others like *EF2* were unreliable [49]. In our study, *UBQ* exhibited the second lowest stability in stems and leaves under cold stress and in roots and stems under heavy metal stress. Regarding environmental conditions, *GAPDH* was identified as the most stable reference gene in *Psammochloa villosa* (Poaceae) under drought stress [50], but it was unstable in *Oryza sativa* (Poaceae) and *Prunus persica* (Rosaceae) under different experimental treatments [51,52,53]. Contrastingly, while *EF-1α* ranks among the most stable genes in drought-treated *S. lycopersicum* and *Arabis alpina* [54,55], it showed the lowest stability across all *R. patientia* tissues under drought. Collectively, these findings demonstrate that stable reference genes vary significantly across species and experimental conditions, highlighting the critical importance of selecting appropriate reference genes for accurate gene expression normalization.

## 5. Conclusions

Comprehensive analysis identified distinct tissue-specific stability patterns among eight candidate reference genes in *R. patientia* subjected to abiotic stresses. *ACT* demonstrated optimal expression stability in roots and leaves under cold stress, stems under drought stress, and roots under salt stress. *SKD1* exhibited superior performance in roots and leaves during drought exposure, all root, stem, and leaf tissues under heavy metal stress, and leaves under salt stress. *TUA* achieved peak stability in stems experiencing both cold and salt stresses. Validation using the drought-responsive *MYB* gene confirmed the reliability of selected reference genes for the seedling tissues analyzed, establishing essential standardization protocols for future transcriptional studies of this species under environmental challenges. However, it is imperative to emphasize that these stability profiles were determined specifically in seedlings. Given the well-documented variation in reference gene stability across developmental stages, future studies are essential to validate and establish appropriate reference genes for mature *R. patientia* plants under these abiotic stresses. Results from this work will benefit future studies on gene expression assessing abiotic stressors in *R. patientia* seedlings and serve as a benchmark for investigations into mature plants.

## Figures and Tables

**Table 1:** Statistical information for primer sequences used to amplify reference genes to normalize RT-qPCR in *R. patientia*.

Gene Name	Gene ID	Primer Sequence (5′-3′) (Forward/Reverse)	Amplicon Length	Tm (°C)	RT-qPCR Efficiency(%)	R^2^
*ACT*	TRINITY_DN197	GGGCTGGCTGATAGGATGAG/ GCACTTGCGATGAACGATT	201	56	95.251	0.999
*GAPDH*	TRINITY_DN2913	AACTTCGGCATTGTTGAGGGAT/ TGGGAATGATGTTGAATGAGGC	127	56	127.364	0.975
*YLS*	TRINITY_DN2081	GTCTTACCTTCTCCCGCATCT/ CAAAGTTCTTTATAGTCTCAGCCAC	164	56	113.547	0.988
*SKD1*	TRINITY_DN115	AGGAAGCCATCACGCAGAAAT/ GCGGAGTTCAACCCAGACCTAA	217	58	101.845	0.998
*UBQ*	TRINITY_DN16	GCTCCCAATACCAACCCTC/ GAATCAGACATAGCACGCAGTT	275	58	97.462	0.992
*UBC*	TRINITY_DN11678	GGACTGACTGGGAAGGTGG/ GACAAACAGTTCCTGATGGGT	132	58	92.583	0.987
*EF-1α*	TRINITY_DN2194	GAAGGAGGCTGCTGAGATG/ GGTGTAGGCAAGAAGAGCG	298	59	108.566	0.986
*TUA*	TRINITY_DN2714	GCTGTGGCTACAATCAAGACC/ AGAACTCACCTTCCTCCATACC	265	59	105.632	0.984

**Table 2:** Expression stability of candidate reference genes from *R. patientia* analyzed by BestKeeper. Abbreviations are as follows: SD, standard deviation; CV, coefficient of variance; C, low-temperature stress; D, drought stress; CD, heavy metal stress; S, salt stress; R, root; ST, stem; L, leaf.

Group	Gene	SD	CV	r	Group	Gene	SD	CV	r	Group	Gene	SD	CV	r
S-R	*ACT*	3.79	12.76	0.983	S-ST	*ACT*	0.98	3.81	0.984	S-L	*ACT*	1.18	4.58	0.126
	*GAPDH*	4.35	16.60	0.993		*GAPDH*	0.74	3.60	0.539		*GAPDH*	0.72	3.28	−0.782
	*YLS*	2.20	7.47	0.978		*YLS*	1.46	5.04	0.87		*YLS*	1.06	3.56	0.729
	*SKD1*	4.01	14.48	0.978		*SKD1*	0.34	1.42	0.461		*SKD1*	0.71	2.89	−0.104
	*UBQ*	3.50	12.46	0.981		*UBQ*	3.37	12.54	0.898		*UBQ*	2.46	8.97	0.876
	*UBC*	1.57	5.81	0.542		*UBC*	2.44	9.43	0.189		*UBC*	1.82	6.92	−0.367
	*EF-1α*	4.67	19.61	0.99		*EF-1α*	4.03	18.66	0.881		*EF-1α*	3.66	16.25	0.675
	*TUA*	5.29	19.23	0.994		*TUA*	0.91	4.35	0.686		*TUA*	4.09	15.84	0.312
D-R	*ACT*	2.97	10.18	0.978	D-ST	*ACT*	1.05	4.01	0.763	D-L	*ACT*	1.13	4.59	0.975
	*GAPDH*	3.13	12.33	0.986		*GAPDH*	0.62	2.94	0.069		*GAPDH*	0.45	2.21	0.278
	*YLS*	1.10	3.65	0.946		*YLS*	1.22	4.25	0.959		*YLS*	1.50	5.39	0.952
	*SKD1*	3.00	10.72	0.993		*SKD1*	0.36	1.48	0.353		*SKD1*	0.96	4.12	0.922
	*UBQ*	1.82	6.32	0.987		*UBQ*	2.33	8.75	0.89		*UBQ*	2.63	10.19	0.945
	*UBC*	0.20	0.73	0.295		*UBC*	2.20	8.49	0.053		*UBC*	2.06	7.89	−0.751
	*EF-1α*	3.91	16.22	0.991		*EF-1α*	3.83	17.33	0.866		*EF-1α*	4.76	21.22	0.972
	*TUA*	3.23	11.99	0.981		*TUA*	0.81	3.84	0.608		*TUA*	0.97	4.52	0.32
C-R	*ACT*	2.77	9.32	0.993	C-ST	*ACT*	0.94	3.54	0.706	C-L	*ACT*	1.25	4.85	0.931
	*GAPDH*	3.72	14.86	0.998		*GAPDH*	0.88	4.17	0.114		*GAPDH*	0.97	4.67	0.408
	*YLS*	1.62	5.38	0.952		*YLS*	1.32	4.55	0.986		*YLS*	1.57	5.48	0.98
	*SKD1*	3.25	11.62	0.986		*SKD1*	0.38	1.52	−0.113		*SKD1*	1.12	4.57	0.558
	*UBQ*	2.6	9.03	0.922		*UBQ*	2.32	8.7	0.861		*UBQ*	2.52	9.77	0.92
	*UBC*	0.25	0.92	0.305		*UBC*	1.94	7.37	−0.134		*UBC*	1.24	4.61	−0.133
	*EF-1α*	3.48	14.9	0.991		*EF-1α*	3.60	15.86	0.797		*EF-1α*	3.89	17.05	0.96
	*TUA*	3.78	14.78	0.936		*TUA*	0.74	3.38	0.13		*TUA*	2.32	10.03	0.982
CD-R	*ACT*	3.63	12.45	0.985	CD-ST	*ACT*	1.6	5.95	0.8	CD-L	*ACT*	1.13	4.410	0.727
	*GAPDH*	3.33	12.93	0.985		*GAPDH*	1.29	5.69	−0.561		*GAPDH*	1.29	5.63	−0.445
	*YLS*	1.36	4.45	0.805		*YLS*	1.38	4.6	0.966		*YLS*	1.77	6.18	0.629
	*SKD1*	3.49	12.48	0.995		*SKD1*	0.46	1.81	−0.371		*SKD1*	0.4	1.63	0.315
	*UBQ*	2.1	7.61	0.95		*UBQ*	2.11	7.78	0.515		*UBQ*	2.94	11.29	0.993
	*UBC*	0.19	0.68	0.494		*UBC*	1.45	5.45	−0.365		*UBC*	1.77	6.71	−0.747
	*EF-1α*	4.24	17.38	0.987		*EF-1α*	3.29	13.57	0.419		*EF-1α*	3.76	16.05	0.914
	*TUA*	3.43	12.37	0.995		*TUA*	1.48	6.08	−0.138		*TUA*	1	4.19	−0.883

**Table 3:** Reference gene ranking using five methods. Abbreviations are as follows: C, low-temperature stress; D, drought stress; CD, heavy metal stress; S, salt stress; R, root; ST, stem; L, leaf; (R), ranking.

Software	Reference Gene	C-R(R)	C-S (R)	C-L (R)	CD-R (R)	CD-S (R)	CD-L (R)	ST-R (R)	ST-S (R)	ST-L (R)	D-R(R)	D-S(R)	D-L(R)
geNorm	*UBC*	8	6	7	8	1	2	8	6	5	8	6	7
	*TUA*	7	1	5	1	4	1	6	1	8	1	3	3
	*YLS*	6	4	4	7	5	6	7	5	4	7	5	5
	*UBQ*	5	7	6	6	7	7	1	7	6	6	7	6
	*ACT*	4	4	3	4	6	5	2	3	2	5	4	4
	*SKD1*	3	3	1	3	3	4	3	4	1	3	1	1
	*GAPDH*	2	2	2	2	2	3	4	2	3	2	2	2
	*EF-1α*	1	8	8	5	8	8	8	8	7	4	8	8
NormFinder	*UBC*	8	7	7	8	5	6	8	7	6	8	7	6
	*TUA*	7	4	3	4	6	4	7	3	8	5	3	8
	*YLS*	5	2	2	7	2	3	6	1	1	6	2	1
	*UBQ*	3	6	5	2	7	7	2	6	5	2	6	5
	*ACT*	1	1	1	3	3	1	1	2	3	3	1	3
	*SKD1*	2	3	4	1	1	2	3	5	2	1	4	2
	*GAPDH*	4	5	6	5	4	5	4	4	4	4	5	4
	*EF-1α*	6	8	8	6	8	8	5	8	7	7	8	7
BestKeeper	*UBC*	1	6	3	1	3	6	1	6	5	1	6	6
	*TUA*	7	2	7	4	6	2	8	4	8	6	3	2
	*YLS*	2	5	4	2	2	5	2	5	3	2	5	5
	*UBQ*	3	7	6	3	7	7	3	7	6	3	7	7
	*ACT*	4	3	5	6	5	3	4	3	4	4	4	4
	*SKD1*	5	1	2	5	1	1	5	1	1	5	1	3
	*GAPDH*	8	4	1	7	4	4	6	2	2	7	2	1
	*EF-1α*	6	8	8	8	8	8	7	8	7	8	8	8
RefFinder	*UBC*	7	6	6	6	4	4	6	7	6	6	7	7
	*TUA*	8	2	5	2	5	3	8	1	8	5	4	5
	*YLS*	6	5	4	7	3	5	5	4	2	7	5	4
	*UBQ*	4	7	7	4	7	7	2	6	5	3	6	6
	*ACT*	1	1	1	5	6	2	1	2	3	4	1	2
	*SKD1*	2	3	3	1	1	1	3	5	1	1	2	1
	*GAPDH*	3	4	2	3	2	6	4	3	4	2	3	3
	*EF-1α*	5	8	8	8	8	8	7	8	7	8	8	8
ΔCt	*UBC*	8	8	8	8	8	8	8	8	8	8	7	6
	*TUA*	4	3	3	5	2	3	4	3	3	2	1	2
	*YLS*	6	7	7	7	7	7	6	6	7	6	8	8
	*UBQ*	2	5	5	3	5	5	2	5	5	3	6	5
	*ACT*	7	6	6	6	6	6	7	6	6	7	4	3
	*SKD1*	1	1	1	1	1	1	1	1	1	4	5	1
	*GAPDH*	5	2	2	2	3	2	5	2	2	5	3	4
	*EF-1α*	3	4	4	4	4	4	3	4	4	1	2	7

## Data Availability

Data will be made available on request.
